# Myopia and Other Visual Disorders in Children

**DOI:** 10.3390/ijerph19158912

**Published:** 2022-07-22

**Authors:** Cristina Alvarez-Peregrina, Clara Martinez-Perez, Miguel Ángel Sánchez-Tena

**Affiliations:** 1 Department of Optometry and Vision, Faculty of Optics and Optometry, Universidad Complutense de Madrid, 28037 Madrid, Spain; cristina_alvarez@ucm.es (C.A.-P.); masancheztena@ucm.es (M.Á.S.-T.); 2 ISEC LISBOA—Instituto Superior de Educação e Ciências, 1750-179 Lisbon, Portugal

Ocular pathologies can lead to permanent vision loss and pose an important public health problem. Globally, it is estimated that around 285 million people have a visual impairment, of whom 19 million are children under the age of 14. In turn, it is estimated that one out of five preschool-aged children has vision problems [1,2].

As vision pathologies become worse over time, an early diagnosis and treatment to avoid long-term vision loss are essential for children. Recognizing ocular pathologies in time is important for children with an intellectual development disorder, as it has been proved that children with disorders have more ocular and vision problems than children without disorders [3,4].

Eye conditions can alter the normal development of children, academic performance, social interactions or self-esteem [5,6,7]. Eighty percent of learning is carried out visually. Thus, children with learning difficulties have more problems linked to vision. These problems are usually related to refractive errors; therefore, an early solution can help children improve their academic performance [8].

Due to the increase in visual problems during childhood and the significant increase in myopia prevalence in school-aged children in recent years, this Special Issue of *IJERPH* aims to include articles and reviews that comprise an extensive repertoire of services and methodological approaches, especially from the perspective of optometry and ophthalmology. Although eye pathologies during childhood are diverse and it is not possible to completely address the matter in this Special Issue, the intention is to include articles that provide information on different key subjects within the pediatrics visual health field.

When carrying out a bibliographic search in the Web of Science database using the search terms “(Ophthalmol* OR Optometry) AND (Pediatric OR Paediatric)” between 1949 June and 2022, 14,447 publications were found. All the publications were analyzed using a citation network analysis, that is to say, one publication is the starting point to find other additional publications that are also relevant with the objective of showing qualitatively and qualitatively the connections between articles and authors through the creation of groups. Moreover, it is also possible to quantify the most cited publications for each group, as well as how to study the development of a research field or prioritize the literature search on a specific subject. Therefore, in this research field, we can highlight eight main groups that analyze eight specific topics. The first group analyses the importance of refractive errors during childhood. The most cited publication in this group had 103 citations. It was published by Donahue et al. [9] in the American Association for Pediatric Ophthalmology and Strabismus in 2013. In this publication, the American Association for Pediatric Ophthalmology and Strabismus Vision Screening Committee suggested detection criteria for amblyopia during the preschool age. Thus, astigmatism over 2.0 D, hypermetropia over 4.5 D and anisometropia over 2.5 D should be detected in children between 12 to 30 months and the same in children aged between 31 to 48 months for astigmatism over 2.0 D, hypermetropia over 4.0 D and anisometropia over 2.0 D. Likewise, for children older than 49 months, astigmatism over 1.5 D, anisometropia over 1.5 D and hypermetropia over 3.5 D should also be detected. Amblyopia is a subject of great interest for researchers because it is the most common visual deficit during childhood. In the United Kingdom, it affects between 2 and 5% of children [10], and it is the second most common reason for low vision in low-income countries [11]. On the other hand, myopia is the most common refractive problem worldwide. It is estimated that 22.9% of the world population is affected by myopia (1406 million people), of whom 2.7% (163 million people) have high myopia. These rates are projected to increase, and by 2050, it is estimated that 49.8% (4758 million people) of the world population will have myopia, and 9.8% (938 million people) will suffer from high myopia [12]. Thus, the financial burden of non-corrected refractive errors, mainly caused by myopia, is estimated to reach 202.000 million dollars a year. The direct cost is related to glasses, contact lenses or refractive surgery. Other medical expenses are related to the treatment of eye complications due to high myopia, such as retinal detachment, myopic macular degeneration, glaucoma and cataracts, as well as the visual impairment and blindness they will lead to. This global cost will continue to increase as the number of people suffering from myopia increases. Patients with a higher myopic refractive error tend to have a lower quality of life and this leads to worse social, economic and health problems. The economic impact, together with the ocular pathologies associated with refractive errors, is the reason why researchers are interested in this topic. In turn, this kind of publication will have a significant impact on this population. Thus, as one of the articles published in this Special Issue notes, on Twitter, there is a mean of 296.36 ± 1585.58 tweets and retweets about myopia [13].

The second group analyzes the prevalence, treatment and complications of congenital cataracts. The most cited article was a paper carried out by Buckley et al. [14], published in the *American Journal of Ophthalmology* in 1993, with 94 citations. This publication discussed a modified standard pediatric cataract surgical procedure based on endocapsular cataract extraction, posterior chamber intraocular lens implantation, posterior pars plana capsulotomy and anterior pars plana vitrectomy. The results found no complications associated with the intraoperative removal of the posterior capsule. Congenital cataracts occur in 1 out of 2000 newborn children, causing 10% of all preventable visual losses in children globally [15]. The decision to carry out surgery is harder in children than in adults, since a subjective visual assessment is not possible for children and surgeons mainly depend on the morphology and location of the cataract, as well as on the child’s behavior. Surgery can be performed within the first three months of life according to experimental and clinical research, since early detection and correction are directly linked to the visual result. There is still controversy regarding the age at which an IOL can be implanted safely in the eye. Aphakia treatment still poses an important problem since glasses or contact lenses are needed. Thus, success is directly linked to the parent´s compliance and the child´s cooperation. The results of pediatric cataract surgery are based not only on anatomical success, but also on the post-surgical course to monitor the clear visual axis and the aggressive management of pre-existing myopia and its prevention [16,17].

The third group analyzed the importance and management of retinal pathologies. The most cited publication was carried out by the International Committee for the Classification of Retinopathy of Prematurity [18], which had 162 citations and it was published in the *Archives of Ophthalmology* in 2005. This article revised the International Classification of Retinopathy of Prematurity. Thus, aspects that differ from the original classification include an introduction to the concept of a more virulent form of retinopathy seen in younger infants and a description of an intermediate level of plus disease between normal posterior pole vessels and frank plus disease and a practical clinical tool to estimate the extent of zone I. Congenital retinal dystrophies can appear at birth or during the first six months of life at the severe end of the spectrum, known as Leber congenital amaurosis. Early severe retinal dystrophia affects young children, compared to juvenile-onset retinitis pigmentosa, which generally affects older children or teenagers when they also have another non-related retinal degeneration problem. Juvenile retinoschisis is linked to chromosome X. The most common macular dystrophies appearing during childhood include Stargardt and Best disease. The symptoms of stationary non-progressive dysfunction of rods and cones, such as achromatopsia or congenital stationary night blindness, are also present during childhood [19].

The fourth group analyzed retinoblastoma. The most cited publication was an article carried out by Dimaras et al. [20], published in the *Nature Reviews Disease Primers* in 2015, with 66 citations. The manuscript included a review on retinoblastoma, which is a rare retinal cancer that affects young children and is diagnosed in approximately 8000 children each year all over the world. It occurs when both alleles of the same retinoblastoma gene (RB1) mutate into a susceptible retinal cell, probably a precursor of the cone photoreceptors. The loss of the retinoblastoma tumor suppressor protein (pRB) leads to uncontrolled cell division and recurrent genomic changes during tumor progression. pRB is expressed in almost all tissues; however, cone precursors have biochemical and molecular characteristics that may be sensitive to the loss of RB1 and cause tumorigenesis. The patient survival rate is >95% in high-income countries, but <30% globally. However, this information has been updated this year. Thus, survival rates are close to 98–99%, and rescue rates for the eyeball are constantly increasing. This can be achieved due to the advances in intra-arterial and intravitreal chemotherapy, which allow us to save the eyes of patients that otherwise would have been lost with more conventional treatment [21].

The fifth group analyzed the treatment and management of glaucoma. The most cited publication was carried out by Beck et al. [22], which was published in the *American Journal of Ophthalmology* in 1998, with 82 citations. This study aimed to determine the safety and effectiveness of trabeculectomy with adjunctive mitomycin C in 17-year-old patients or younger and identify the risk factors that cause this surgical technique to fail. The results showed that this technique is effective for pediatric glaucoma treatment, especially in phakic children older than a year. Of note, late-onset blister-related endophthalmitis was a common risk in these patients. “Infantile glaucoma” is a rare and serious condition that often leads to significant visual loss. It is a group of heterogeneous diseases characterized by elevated intraocular pressure (IOP) and damage to the optic disc. Good IOP control is essential, but in most patients, it is achieved surgically along with medical therapy. Currently, there are various classifications of childhood glaucoma, but it can be simply classified as primary (there is an abnormality in the development of the anterior chamber angle) and secondary (aqueous discharge is reduced due to independent mechanisms that secondarily affect the function of the anterior chamber angle). Primary glaucoma in children is usually classified based on the age of onset: primary congenital glaucoma, from birth to early childhood, and primary juvenile open-angle glaucoma, from age 4 to early adulthood. Secondary glaucoma includes a variety of conditions that are a result of damage to the aqueous humor fluid drainage system due to congenital or acquired eye diseases or systemic disorders [23].

The sixth group analyzed idiopathic intracranial hypertension. The most cited publication was carried out by Rangwala et al. [24], with 79 citations, published in the *Survey of Ophthalmology* in 2007. The authors carried out a review on said pathology. The incidence of idiopathic intracranial hypertension appears to be increased in adolescent children. In older children, it is clincally similar to that of idiopathic intracranial hypertension in adults (women and obese). In the younger age group, non-obese children may develop idiopathic intracranial hypertension. However, the pathogenesis of the disease is still unknown. In young children, this pathology is associated with several new etiologies, including recombinant growth hormone and all-trans retinoic acid. To exclude intracranial processes, more modern neuroimaging techniques are used, such as magnetic resonance imaging and magnetic resonance venograms. Although most cases of pediatric idiopathic intracranial hypertension improve with medical treatment, those who have achieved visual progression despite medical treatment have undergone optic nerve sheath fenestration and lumboperitoneal bypass.

The seventh group analyzed the importance of ocular lesions. The most cited publication was carried out by Strahlamn et al. [25], which had 65 citations and was published in the *Archives of Ophthalmology* in 1990. In this publication, the incidence of ocular trauma in Maryland children was estimated (at 15.2 per 100,000 per year). Male patients outnumbered female patients as victims of eye lesions by a ratio of 4:1; eye lesions were two times more likely to occur in children aged between 11 and 15 years old than in younger children. The most common cause of pediatric ocular trauma was accidental blows and falls (37%). Sports and leisure activities accounted for 27% of all ocular lesions, 39% of all non-penetrating lesions and 40% of all lesions in children aged between 11 and 15 years old. Other important causes of eye lesions were burns (9%), car accidents (11%) and non-powder firearms accidents (4%). It should be highlighted that ocular lesions in children are one of the main causes of acquired blindness. The consequences of childhood lesions are harmful as they lead to permanent blindness and visual impairment. They also affect the psychological, social and emotional development of the child. Likewise, parents are also worried about the child´s future. Globally, ocular trauma amongst children represents 8% to 14% of total lesions [26]. Childhood ocular lesions are different from adulthood ocular trauma in terms of the objects involved in the lesion, the assessment and the management protocols. Thus, it includes abrasions on the corneal surface to corneal and scleral perforations. Most ocular lesions in children are preventable by taking minor precautions and identifying the risk factors for the ocular lesion.

The eighth group analyzed the incidence and treatment of uveitis. The most cited publication was carried out by Smith et al. [27], which had 102 citations and was published in *Ophthalmology* in 2009. A total of 526 children with a mean age of 9 years old who were diagnosed with uveitis participated in this study. The main diagnosis was idiopathy (28.8%), uveitis associated with juvenile idiopathic arthritis (20.9%) and pars planitis (17.1%). The insidious onset (58%) and persistent duration (75.3%) were the most common; anterior uveitis was the most predominant (44.6%). Complications were frequent and cystoid macular edema had the most significant visual impact. Uveitis in the pediatric population is not common as it represents 2 to 14% of all uveitis cases, but it results in significant ocular morbidity. The course of the disease has a high complication rate that may lead to a person becoming blind and impaired for life. Challenges in the management of pediatric uveitis in young people are mainly related to a late diagnosis either because they are unable to verbalize their complaints or because the disease itself may not present any symptoms. Pediatric uveitis has specifications related to aetiologies, such as juvenile idiopathic arthritis and Kawasaki´s disease, or it also has later complications such as amblyopia. Juvenile idiopathic arthritis is mainly associated with pediatric uveitis and it is the main cause of visual loss in children according to most previous studies. Amblyopia caused by prolonged inflammation or its consequences is associated with the pediatric group that required early and aggressive intervention [28,29,30]. Thus, there is a need for increased research in order to better diagnose and manage multiple ocular pathologies; therefore, this Special Issue is an invitation to publish articles that will address the research fields that have been analyzed in this Editorial.
